# Inherited MST1 Deficiency Underlies Susceptibility to EV-HPV Infections

**DOI:** 10.1371/journal.pone.0044010

**Published:** 2012-08-27

**Authors:** Amandine Crequer, Capucine Picard, Etienne Patin, Aurelia D’Amico, Avinash Abhyankar, Martine Munzer, Marianne Debré, Shen-Ying Zhang, Geneviève de Saint-Basile, Alain Fischer, Laurent Abel, Gérard Orth, Jean-Laurent Casanova, Emmanuelle Jouanguy

**Affiliations:** 1 St. Giles Laboratory of Human Genetics of Infectious Diseases, Rockefeller Branch, The Rockefeller University, New York, New York, United States of America; 2 Paris Descartes University, Sorbonne Paris Cité, Necker Medicine Faculty, Paris, France; 3 Laboratory of Human Genetics of Infectious Diseases, Necker Branch, INSERM U980, Paris, France; 4 Study Center for Primary Immunodeficiencies, Necker Hospital, AP-HP, Paris, France; 5 Human Evolutionary Genetics, CNRS URA 3012 Genomes and Genetics Department, Pasteur Institute, Paris, France; 6 Department of Pediatrics, American Memorial Hospital, Centre Hospitalier Regional et Universitaire (CHU) de Reims, Reims, France; 7 Pediatric Immuno-hematology Unit, Necker Hospital, AP-HP, Paris, France; 8 Laboratory of Normal and Pathological Development of the Immune System INSERM U768, Paris, France; 9 Department of Virology, Pasteur Institute, Paris, France; University of Washington, United States of America

## Abstract

Epidermodysplasia verruciformis (EV) is characterized by persistent cutaneous lesions caused by a specific group of related human papillomavirus genotypes (EV-HPVs) in otherwise healthy individuals. Autosomal recessive (AR) EVER1 and EVER2 deficiencies account for two thirds of known cases of EV. AR RHOH deficiency has recently been described in two siblings with EV-HPV infections as well as other infectious and tumoral manifestations. We report here the whole-exome based discovery of AR MST1 deficiency in a 19-year-old patient with a T-cell deficiency associated with EV-HPV, bacterial and fungal infections. MST1 deficiency has recently been described in seven patients from three unrelated kindreds with profound T-cell deficiency and various viral and bacterial infections. The patient was also homozygous for a rare *ERCC3* variation. Our findings broaden the clinical range of infections seen in MST1 deficiency and provide a new genetic etiology of susceptibility to EV-HPV infections. Together with the recent discovery of RHOH deficiency, they suggest that T cells are involved in the control of EV-HPVs, at least in some individuals.

## Introduction

Epidermodysplasia verruciformis (EV) is a rare genodermatosis characterized by persistent cutaneous lesions caused by a specific group of related human betapapillomavirus genotypes (EV-HPVs) in otherwise healthy individuals [1], [2]. EV is also associated with a high risk of skin cancer. Many cases of EV are familial, and 10% of patients come from consanguineous families [2]. This observation led Cockayne *et al.* to hypothesize in 1933 that EV resulted from autosomal recessive (AR) inborn errors of immunity against EV-HPVs [3]. This hypothesis was confirmed by the discovery of the first two genetic etiologies of EV in 2002, mutations in *EVER1* and *EVER2* [4], which added EV to the list of primary immunodeficiencies [5]–[8]. These genes, which are expressed in both keratinocytes and leukocytes, are involved in the control of intracellular zinc distribution [9]. The inactivating mutations in *EVER1* or *EVER2* discovered to date account for approximately 75% of all cases reported worldwide [1], [2], [10]–[16]. We recently identified two siblings with RHOH deficiency, EV-HPV infections, other cutaneous viral infections, a bronchopulmonary disease of unclear pathogenesis, and a Burkitt lymphoma [17]. These siblings do not display EV *stricto sensu*. Consistent with the patients’ broad phenotype, RHOH is found specifically in leukocytes, including T cells, but is absent from keratinocytes. RHOH-deficient patients have a T-cell defect characterized by abnormally low numbers of naive T cells, high numbers of memory T cells and impaired T-cell proliferation in response to TCR stimulation. The discovery of two RHOH-deficient patients suggests an important role for T cells in immunity to EV-HPVs, at least in some individuals. Of note, the T-cell defect in these two siblings also accounts for the development of infectious and tumoral signs other than EV-HPV infections [17]. In addition to inherited susceptibility to EV-HPV infections, an acquired form has been described in patients with HIV infection [18], [19], renal allograft transplant recipients [18], [20] and in patients undergoing chemotherapy [18], [21] or hematopoietic stem cell transplantation (HSCT) for severe combined immunodeficiency (SCID) caused by mutations in *JAK3* or *IL2RG,* which encodes the common gamma chain (γc) [18], [22]. Intriguingly, EV-HPV lesions have not been reported after HSCT in patients with other inborn errors of immunity, suggesting that residual JAK3 and IL2RG deficiencies in keratinocytes may be involved in the pathogenesis of this condition [22]. Here, based on whole-exome sequencing (WES), which has successfully been used to identify novel immune deficiencies [23]–[25], we describe the discovery of MST1 deficiency and a profound T-cell deficiency in a patient displaying EV-HPV infections as well as candidiasis and pulmonary infections.

## Results

### Case Report

**Figure 1:**
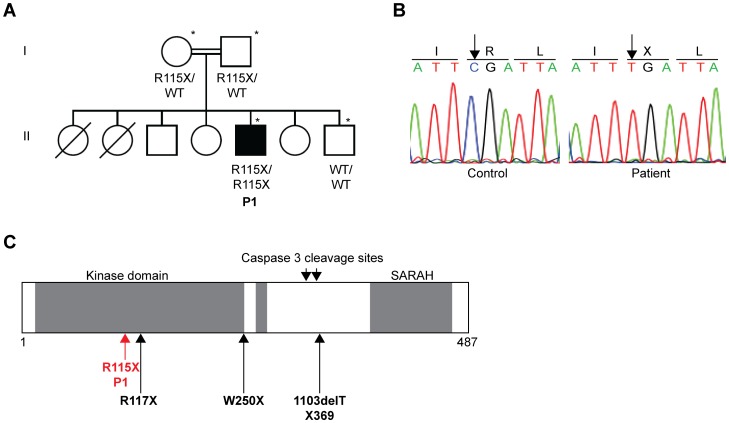
Homozygous *MST1* nonsense mutation in one patient with EV-HPV, bacterial and fungal infections. (A) Pedigree of the family with EV-HPV, bacterial and fungal infections. Generations are designated by a Roman numeral (I, II). P1 is represented by a black symbol. The symbol *indicates the individuals genotyped with the Affymetrix Genome-wide SNP 6.0 array. The Familial segregation of the mutation R115X is shown on the pedigree. (B) Automated sequencing profile, showing the R115X *MST1* mutation in gDNA extracted from EBV-B cells from the patient and comparison with the sequence obtained from a healthy control. The C→T mutation leads to the replacement at residue 115 of an Arg (R) residue by a STOP codon (X). (C) Schematic representation of the structure of the MST1 protein adapted from the work of Nehme *et al.* [30]. R115X is situated in the kinase domain, close to the previously reported R117X mutation described by Nehme *et al.,* indicated by a black arrow. The second mutation (1103delT X369) described by Nehme *et al.,* 1103delT X369, and the mutation described by Abdollahpour *et al.* (W250X) [31] are also indicated by black arrows.

The patient (P1) was born to first-cousin parents of Senegalese origin living in France (Figure 1A). He had a total of six siblings, four of whom are alive and healthy. His eldest sister died from a febrile cough at the age of five months. The second eldest sister died at the age of 18 years, from unknown causes, shortly after giving birth. At one year of age, P1 presented bronchitis requiring a short period of hospitalization. He subsequently had chicken-pox, with no complications. At the age of six years, he began to develop disseminated flat warts, mostly on the face, behind the ears, and on the upper and lower extremities. The skin lesions extended to the chest, the back and the perianal zone over the next few years of his life. During this period, the patient also developed lesions in the mouth, on the inner part of the lips and cheeks, and on the tongue. The skin lesions were found to be caused by EV-HPVs: HPV5, HPV15 and a third unclassified type by Southern Blot and PCR (data not shown). At the age of 13 years, the patient was hospitalized for severe pneumonia requiring intensive care and antibiotic treatment. He displayed no cardiac clinical phenotype and no electrocardiogram or echocardiographic abnormalities. He had Epstein Barr virus (EBV) infection, without severe symptoms, at the age of 14 years (1,500 copies/ml, determined by PCR). At the same age, the patient suffered from major tooth cavities and developed submental cervical adenopathy. The cervical lymph nodes were consequently removed. Histological analysis of one of the lymph nodes removed showed a fibrous thickening of the capsule associated with an increase in the abundance of plasmocytes and the presence of a few HHV8^+^ cells and a few cells labeled with the Epstein-Barr-encoded RNA (EBER) probe. Since the age of 15, white patches testing positive for *Candida albicans* have regularly been observed in his mouth and treated with Fluconazole. At the age of 17, signs of autoimmunity were detected, with the presence of autoantibodies against proteinase 3 (cANCA) in the patient’s serum (Table S1). All classical vaccines administered during childhood – the live vaccines BCG (Monovax®), measles, mumps and rubella and the surface antigen vaccines diphtheria, tetanus toxoid, poliovirus and HBV – were carried out without complications, but PPD tests have remained negative. At 10 years old, the patient’s growth curve presented a negative deviation from the norm. At the age of 17, stabilization at −2SD (standard deviation) for weight and −1SD for height was observed. The patient is now 19 years old and has been receiving immunoglobulin (Ig) replacement therapy since the age of 17 and antibiotic prophylaxis (Trimethoprim/Sulfamethoxazole) against infection since the age of 13.

### Immunological Explorations

We carried out a general immunophenotyping of the patient’s peripheral blood cells at various ages, from nine to 19 years. The number of peripheral polymorphonuclear neutrophils gradually decreased over time, whereas monocyte counts were normal. The proportion of CD3^+^ T cells was slightly lower than the normal range for age, from the age of 13 years onwards. This general T-cell lymphopenia was associated with more pronounced CD4^+^ T-cell lymphopenia (Table 1). CD8^+^ T-cell counts and proportions were normal or higher than control values, depending on the experiment. The Vαβ repertoire of CD8^+^ T cells had an abnormal distribution, with an excess of the Vβ14 subset (data not shown). Within the CD4^+^ T-cell compartment, CD45RO^+^ memory cells were present at a much higher level and CD45RA^+^ naive T cells were much less frequent than normal (Table 1). Within the naive subset, recent thymic T-cell emigrants (CD31^+^CD45RA^+^CD4^+^) were almost entirely absent in analyses of the patient’s peripheral blood (Table 1). We then studied the frequencies of the four functionally different types of CD8^+^ T lymphocytes defined on the basis of CD45RA and CCR7 expression [26], when the patient reached 19 years of age. There were very few naive CD8^+^ T cells (CCR7^+^CD45RA^+^CD8^+^), the percentage of these cells being well below the normal range. The percentage of central memory CD8^+^ T cells (CCR7^+^CD45RA^−^CD8^+^) was also lower than normal, whereas the proportion of cells belonging to the effector memory T_EM_ (CCR7^−^CD45RA^−^CD8^+^) subset was normal. By contrast, the revertant memory T_EMRA_ (CCR7^−^CD45RA^+^CD8^+^) subset was more abundant than in controls (Table 1). The frequencies of B and NK cells were normal (Table 1).

**Table 1:** Immunophenotyping of the patient.

	Patient (normal range)^A^				
Patient’s age	9 years	13 years	16 years	17 years	19 years
Neutrophils (×10^9^/l)	2.0 (1.5–3.6)	**1.0** (1.5–3.5)	ND	**0.8** (1.5–3.5)	**1.0** (1.5–3.5)
Eosinophils (×10^9^/l)	0.4 (≤0.8)	ND	ND	0.1 (≤0.8)	0.1 (≤0.8)
Monocytes (×10^9^/l)	0.6 (0.24–0.75)^B^	ND	ND	0.5 (0.21–0.73)^B^	0.6 (0.21–0.73)^B^
*Total lymphocytes (counts/µl)*	4,400 (1,900–3,700)^C^	**900** (1,900–3,700)^C^	**600** (1,400–3,300)^C^	**700** (1,400–3,300)^C^	**900** (1,400–3,300)^C^
*T cells*					
CD3^+^ (%)	**48** (60–76)^D^	51 (56–84)^D^	73 (56–84)^D^	74 (56–84)^D^	**62** (64–85)^E^
CD3^+^ counts *(counts/µl)*	2112 (1,200–2,600)^D^	**459** (1,000–2,200)^D^	**438** (1,000–2,200)^D^	**518** (1,000–2,200)^D^	**558** (807–1844)^E^
CD4^+^ (%)	**12** (31–47)^D^	**18** (31–52)^D^	**19** (31–52)^D^	**27** (31–52)^D^	37 (34–62)^E^
CD4^+^ counts *(counts/µl)*	**528** (650–1,500)^D^	**162** (530–1,300)^D^	**114** (530–1,300)^D^	**189** (530–1,300)^D^	**333** (460–1232)^E^
CD8^+^ (%)	48 (18–35)^D^	43 (18–35)^D^	49 (18–35)^D^	39 (18–35)^D^	25 (14–42)^E^
CD8^+^ counts *(counts/µl)*	2112 (370–1,100)^D^	387 (330–920)^D^	**294** (330–920)^D^	**273** (330–920)^D^	225 (187–844)^E^
CD4^+^CD45RA^+^ (%)	ND	16 (33–66)^D^	12 (33–66)^D^	9 (33–66)^D^	**7** (58–70)
CD4^+^CD45RO^+^ (%)	ND	84 (18–38)^D^	88 (18–38)^D^	91 (18–38)^D^	93 (16–34)^E^
CD4^+^CD31^+^CD45RA^+^ (%)	ND	**7** (42–74)^C^	**7** (42–55)^C^	**4** (42–55)^C^	**3** (43–55)
CD8^+^CCR7^+^CD45RA^+^ (%)	ND	ND	ND	ND	**4** (37–50)
CD8^+^CCR7^+^CD45RA^−^ (%)	ND	ND	ND	ND	**0.4** (6–16)
CD8^+^CCR7^−^CD45RA^+^ (%)	ND	ND	ND	ND	71 (8–20)
CD8^+^CCR7^−^CD45RA^−^ (%)	ND	ND	ND	ND	24 (24–37)
TcRγδ (%) (2–8^A^)	ND	13	ND	ND	ND
*B cells*					
CD19^+^ (%)	9 (13–27)^D^	14 (6–23)^D^	9 (6–23)^D^	11 (6–23)^D^	10 (6–17)^E^
CD27^+^CD19^+^ (%) (>15^A^)	ND	ND	27	ND	30
*NK cells*					
CD56^+^CD3^+^ (%)	ND	12	13	ND	ND
CD56^+^CD3^−^ (%)	ND	28 (6–27)^F^	14 (6–27)^F^	ND	ND
CD16^+^CD56^+^ (%)	ND	ND	ND	16 (3–22)	22 (3–22)

A Normal ranges were obtained from internal laboratory controls (N = 10) unless specified otherwise.

B normal ranges from the work of Lugada *et al.* [47].

C normal ranges taken from the work of Nehme *et al.* [30].

D normal ranges taken from the work of Shearer *et al.* [48].

E normal ranges taken from the work of Bisset *et al.* [49].

F normal ranges taken from the work of Eidenscheck *et al.* [50].

We also investigated the proliferative response of T cells to various mitogens and antigens *in vitro* (Table 2). The patient’s T cells consistently responded poorly to mitogens (PHA, PMA + ionomycin) and the anti-CD3 antibody OKT3 and did not respond at all to recall antigens, despite normal immunization (tetanus anatoxin, PPD) or prior infection (*C. albicans*). The patient also had moderately high IgG levels, but very high levels of IgA and IgE (Table S1). Despite this hypergammaglobulinemia, the patient displayed a poor antibody response to several immunizations: no antibodies were produced against diphtheria, pneumococcus or *Haemophilus influenzae* and no allohemagglutinins against blood group antigens were detected. However, the patient did generate detectable amounts of antibodies against rubella and varicella zoster virus (VZV). The overall immunological features of the patient were consistent with a T-cell immunodeficiency.

**Table 2:** T-cell proliferation in response to mitogens and antigens, as assessed by thymidine incorporation^A^.

Stimulus (normal range)^B^	Patient (P1)				
Patient’s age	9 years	13 years	14 years	16 years	18 years
*3-day culture*					
PHA (>50)	49	18	22	18	58.5
OKT3 (50 ng/ml ) (>30)	10.6	17	24	ND	ND
PMA (10^−7^M) + Ionomycin (10^−5^M) (>80)	ND	ND	14	ND	ND
PMA (10^−8^M) + Ionomycin (10^−6^M) (>80)	ND	ND	0.10	ND	ND
*6-day culture*					
Tetanus toxoid (>10)	0	1	0	0.2	1.4
Tuberculin (>10)	ND	6	ND	0.4	ND
Candidin (>10)	1.2	2.2	2	0.1	1.2
HSV (>10)	ND	ND	ND	0.1	0.5

A 3H-TdR incorporation in cpm/10^3^.

B normal ranges from internal laboratory controls.

### Genetics

MHC class II deficiency was excluded, because HLA-DR expression on CD19^+^ and CD14^+^ cells was normal. Mutations of *EVER1*, *EVER2*, *IL2RG*, *JAK3*, *CXCR4, RAG1, RAG2, ARTEMIS, TYK2* were previously excluded by homozygosity mapping or direct sequencing. As the patient was born to consanguineous parents, we hypothesized that his immunodeficiency was inherited as an AR trait. We thus performed a genome-wide scan with the Affymetrix genome-wide SNP 6.0 array, followed by a multipoint genome-wide linkage (GWL) analysis with homozygosity mapping, using a complete penetrance model. We identified 17 regions on 11 chromosomes with a maximum LOD score of 1.33 (Figure S1). These regions covered a total of 81.7 Mb, containing 1,254 genes, including 707 protein-coding genes. We then carried out WES for the patient, covering 705 protein-coding genes in the linked regions (the *TMEM185B* gene encoding the transmembrane protein 185 B and *MTRNR2L2* encoding the MT-RNR2-like 2 protein were not covered) (data not shown) with a mean of 20.9 reads per gene. We identified 9,084 coding variations, after alignment and variant calling, 450 of which were not reported in the NCBI dbSNP 134 and the 1000 Genomes (http://www.1000genomes.org/) databases (Table 3). Seven of these variations were in the linked regions, and six were homozygous. Among those six homozygous variants, only two were not present in our in-house database (composed of 615 exomes from patients with different clinical phenotypes): the I285V variant of the *ERCC3* gene (located in region 2q21) and the R115X variant of the *STK4/MST1* gene (located in region 20q11.2–q13.2).

**Table 3:** Whole-exome analysis of P1^a^.

	Total coding	Coding in 1kgenomesand dbSNP134	Novel coding	Total essential splice	Novel essential splice	Total ncRNA	Novel ncRNA
Whole Exome	9,084	8,634	450	340	8	840	39
Linked regions	258	251	7	12	0	18	0

Coding variants include missense, nonsense, frameshift, in-frame deletions and insertions and readthrough variants.

Essential splice variants include all variants found in the first two base pairs or the last two base pairs of introns.

a Both homozygous and heterozygous variations are included.

The *xeroderma pigmentosum type B (XPB)* gene (*ERCC3*) encodes the excision repair cross-complementing rodent repair deficiency complementation group 3 protein, a DNA helicase in nucleotide excision repair that is also a subunit of a class II transcription factor. It is expressed widely, including in keratinocytes [27], [28]. Mutations of this gene have been found in patients suffering from xeroderma pigmentosum (XP) with or without Cockayne syndrome (CS) and in two patients suffering from trichothiodystrophy (TTD) [29]. Of note, XP, CS and TTD are autosomal recessive inherited disorders [29]. All patients with these conditions display sun sensitivity, often associated with a high susceptibility to skin cancers on areas of the body exposed to the sun and developmental or neurological features. Patients with CS also often have a short stature. In general, these patients neither present susceptibility to infections nor immunological defects [29]. The I285V variant is located in the DNA recognition domain and is predicted to be “tolerated” by SIFT (http://sift.jcvi.org) and “benign” by Polyphen2 (http://genetics.bwh.harvard.edu/pph2/). However, this mutation is unlikely to account for the T-cell and EV-HPV infections of this patient, as these phenotypes have never before been reported in any of the nine patients with ERCC3 deficiency investigated [29], although it may have contributed to the growth retardation of P1. Moreover, none of the other typical phenotypic traits of ERCC3 deficiency were documented in the patient.

MST1 (or STK4, serine/threonine kinase 4) deficiency was recently described by Nehme *et al.* [30] and Abdollahpour *et al.* [31] in seven patients from three unrelated kindreds with a T-cell phenotype closely resembling that of P1. MST1 deficiency in humans has been shown to lead to CD4^+^ T-cell lymphopenia, with naive CD4^+^ and CD8^+^ T-cell lymphopenia, high exhausted activated/memory CD8^+^ T-cell counts and impairment of the response of T cells to stimulation with CD3 antibodies, various mitogens and recall antigens [30]. It has also been associated with neutropenia [31] and heart malformations in some patients [31]. Furthermore, recurrent pulmonary infections, susceptibility to candidiasis and non regressing cutaneous warts caused by cutaneous alpha- and beta-HPVs have been reported in MST1-deficient patients [30], [31]. However, the beta-HPV infected skin lesions observed in one patient were histologically different from the typical EV-HPV infected lesions observed in our patient and EV patients [31]. Strictly speaking, none of the seven previously reported MST1-deficient patients displayed EV-HPV infections, even in a form associated with other infectious and tumoral signs (distinct from EV, which is defined by such lesions in otherwise healthy individuals). The homozygous R115X nonsense mutation of *MST1* was confirmed by Sanger sequencing (Figure 1B). It is located upstream from the previously reported mutations of this gene (Figure 1C), including the loss-of-expression mutation R117X. Methionine-driven translation re-initiation between codons 115 and 117 is not possible. The R115X allele segregated with the disease, with a complete penetrance, consistent with the results of GWL analysis. It therefore is highly likely that the mutation of the *MST1* gene found here is loss-of-expression and disease causing in this patient. All clinical manifestations in P1, except for the growth retardation, and all immunological anomalies, T cells anomalies in particular, can be explained by MST1 deficiency. The infectious diseases seen in some but not all MST1-deficient patients, including susceptibility to EV-HPV lesions, may reflect clinical exposure or the impact of modifier genes. The lack of cardiac anomaly in P1, like in 4 other MST1-deficient patients [30], may also reflect the impact of modifier genes or other genetic lesions in the 3 MST1-deficient patients with such anomalies [31].

## Discussion

We report here the first patient with susceptibility to EV-HPV lesions due to MST1 deficiency. All patients with MST1 deficiency have a similar immunological phenotype, characterized by naive CD4^+^ and CD8^+^ T-cell lymphopenia in particular. Nehme *et al.* showed that MST1 deficiency was associated with an impairment of naive T-cell (CD4^+^ and CD8^+^) survival and impairment of the homing of CD8^+^ cells to secondary lymphoid organs, due to weak expression of the homing receptors CCR7 and CD62L [30]. This homing defect is consistent with findings in the mouse model, in which MST1 deficiency leads to naive T-cell lymphopenia and an impaired egress of mature T lymphocytes from the thymus to secondary lymphoid organs, associated with an impaired chemotactic response to several chemokines, including the CCR7 ligands CCL19 and CCL21 [32], [33]. However, the infectious phenotype differs between patients. Previously reported patients displayed recurrent bacterial infections of the lower respiratory tract leading to bronchiectasis, recurrent HPV, HSV, VZV and *Molluscum contagiosium* infections of the skin, mucocutaneous candidiasis (CMC) and chronic EBV infections [30], [31]. All but one of these patients had had multiple infections by the age of four years. The remaining patient did not display any symptoms of the condition until the age of 10 years. By contrast, our patient presented recurrent pulmonary infections and recurrent oral candidiasis, but his cutaneous phenotype was limited to warts caused by beta-HPV infections, different from the cutaneous lesions observed in other patients with warts caused by other HPV infections [4]. MST1 deficiency is therefore a genetic etiology of susceptibility to EV-HPVs. This experiment of nature suggests that T cells may be critical for protective immunity against cutaneous EV-HPVs, at least in some individuals, which is consistent with our recent discovery of RHOH deficiency in two siblings with EV-HPV lesions and other phenotypes [17]. Nevertheless, there is growing evidence in the mouse model that MST1, which was initially identified as a member of the Hippo tumor-suppressor pathway, negatively regulates cell proliferation and promotes cell differentiation by inactivation of the yes-associated protein, YAP, in various tissues, including the skin [34]–[36]. Inactivation of YAP alleviates its inhibition of the Notch transcriptional pathway, which is known to play an antiproliferative effect in keratinocytes and has recently been shown to be negatively regulated by the beta-HPV protein E6 [37]. We therefore cannot rule out the possibility that MST1 plays a role similar to that of the EVER proteins in restricting viral replication in keratinocytes by keeping cell proliferation rates low. Further studies are required to decipher the cellular pathogenesis of susceptibility to EV-HPV infection, in patients with EV and mutations in *EVER1* or *EVER2*, and in patients with EV-HPV lesions in the context of other phenotypes and mutations in *RHOH* or *MST1*.

## Materials and Methods

### Ethics Statement

This study was conducted in accordance with the Helsinki Declaration, with written informed consent obtained from each patient or the patient’s family. Approval for this study was obtained from the French IRB (Comité de protection des personnes or CPP), INSERM and the Rockefeller IRB.

### Genotyping and Linkage Analysis

Genomic DNA was isolated from lymphoblastoid cell lines or whole-blood samples, by phenol/chloroform extraction. The two parents, the patient and his healthy sibling were genotyped with the Affymetrix Genome-wide SNP 6.0 array. Genotype calling was achieved with Affymetrix Power Tools (http://www.affymetrix.com/partners_programs/programs/developer/tools/powertools.affx) for the four family members and for an additional sample of 200 individuals genotyped by the same platform, to improve the detection of genotype clusters. We discarded monomorphic SNPs, SNPs with a call rate lower than 100% and SNPs presenting Mendelian inconsistencies within the family. SNPs were further filtered with population-based filters. We then used about 87,300 high-quality SNP markers to carry out linkage analysis by homozygosity mapping, assuming autosomal recessive inheritance with complete penetrance. Parametric multipoint linkage analysis was carried out with the Merlin program [38]. The Senegalese family founders and HapMap YRI trios were used to estimate allele frequencies and to define linkage clusters, with an *r^2^* threshold of 0.4. We searched for homozygous deletions in patients, with PennCNV-joint [39], correcting for waviness. P1 presented no homozygous deletion within the linkage regions that encompassed known coding genes and was absent from the DGV database (http://projects.tcag.ca/variation/) (data not shown).

### Sequencing

Polymerase chain reaction (PCR) was carried out with *Taq* polymerase (Invitrogen, Carlsbad, California, USA) and the GeneAmp PCR System 9700 (Applied Biosystems, Foster City, California, USA). Primer sequences are available upon request. The PCR products were sequenced with the BigDye Terminator Cycle sequencing kit (Applied Biosystems). Sequencing products were purified by centrifugation through Sephadex G-50 Superfine resin and sequences were analyzed with a 3730 DNA Analyzer (Applied Biosystems).

### Whole-exome Sequencing

Exome capture was performed with the Agilent SureSelect Human All Exon 50 Mb kit (Agilent Technologies). Paired-end sequencing was performed on an Illumina HiSeq 2000 (Illumina) generating 100-base reads. We aligned the sequences with the hg19 reference build of the human genome, using the BWA aligner [40]. Downstream processing and variant calling were carried out with the Genome Analysis Toolkit (GATK) [41], Samtools [42] and Picard (http://picard.sourceforge.net). Substitution and InDel calls were made with GATK UnifiedGenotyper. All calls with a read coverage <2× and a phred-scaled SNP quality of 20 were filtered out. Variants were annotated with GATK GenomicAnnotator.

### Antibodies

The anti-CD3 mAb OKT3 (IgG2a) has been described elsewhere [43]. Immunologic analysis of the T-, B-, and NK cell compartments of whole-blood samples was performed by flow cytometry with monoclonal antibodies against CD3, CD4, CD8, CD19, CD16, CD45RA, CD45RO and CD31 (Becton Dickinson), as described elsewhere [44]–[46].

### Thymidine Incorporation Assay

PBMCs were incubated for three days alone or with PHA (2.5 µg/ml), or the monoclonal soluble anti-CD3 antibody OKT3 (10, 25, 50 ng/ml), or PMA (10^−7^ or 10^−8^ M) plus ionomycin (10^−5^ or 10^−6^ M), or for six days with tetanus toxoid (0.2–0.4 µg/ml), HSV, tuberculin (5 µg/ml) or candidin (50 µg/ml). Cultures were pulsed with tritiated thymidine for the last 18 hours of the incubation period. The radioactivity incorporated was determined with a Matrix 96 beta counter (Canberra Packard, Frankfurt/Main, Germany). Cell proliferation was assessed by determining the cpm for [^3^H] thymidine incorporation, as previously described [45], [46].

## Supporting Information

Figure S1 **Analysis of multipoint linkage between the patient’s disease and chromosomes 1, 2, 3, 4, 5, 8, 11, 12, 14, 16, 20, with a full penetrance model.** Only chromosomes including regions with a maximal LOD score are shown. LOD scores (Y axis) are plotted against chromosomal position (in cM). The locations of *ERCC3* and *MST1* are indicated by black arrows.
(TIF)

Table S1 Humoral immunity of the patient’s peripheral blood before Ig substitution.
(DOCX)

## References

[1] Orth G (2006) Genetics of epidermodysplasia verruciformis: Insights into host defense against papillomaviruses. Semin Immunol 18: 362–374. 17011789 10.1016/j.smim.2006.07.008

[2] Orth G (2008) Host defenses against human papillomaviruses: lessons from epidermodysplasia verruciformis. Curr Top Microbiol Immunol 321: 59–83. 18727487 10.1007/978-3-540-75203-5_3

[3] Cockayne E (1933) Inherited abnormalities of the skin and its appendages. Oxford University Press, London.

[4] Ramoz N, Rueda LA, Bouadjar B, Montoya LS, Orth G, et al (2002) Mutations in two adjacent novel genes are associated with epidermodysplasia verruciformis. Nat Genet 32: 579–581. 12426567 10.1038/ng1044

[5] Casanova JL, Abel L (2005) Inborn errors of immunity to infection: the rule rather than the exception. J Exp Med 202: 197–201. 16027233 10.1084/jem.20050854 PMC2212996

[6] Casanova JL, Abel L (2007) Primary immunodeficiencies: a field in its infancy. Science 317: 617–619. 17673650 10.1126/science.1142963

[7] Alcais A, Abel L, Casanova JL (2009) Human genetics of infectious diseases: between proof of principle and paradigm. J Clin Invest 119: 2506–2514. 19729848 10.1172/JCI38111 PMC2735901

[8] Alcais A, Quintana-Murci L, Thaler DS, Schurr E, Abel L, et al (2010) Life-threatening infectious diseases of childhood: single-gene inborn errors of immunity? Ann N Y Acad Sci 1214: 18–33. 21091717 10.1111/j.1749-6632.2010.05834.x

[9] Lazarczyk M, Pons C, Mendoza JA, Cassonnet P, Jacob Y, et al (2008) Regulation of cellular zinc balance as a potential mechanism of EVER-mediated protection against pathogenesis by cutaneous oncogenic human papillomaviruses. J Exp Med 205: 35–42. 18158319 10.1084/jem.20071311 PMC2234378

[10] Aochi S, Nakanishi G, Suzuki N, Setsu N, Suzuki D, et al (2007) A novel homozygous mutation of the *EVER1/TMC6* gene in a Japanese patient with epidermodysplasia verruciformis. Br J Dermatol 157: 1265–1266. 17916203 10.1111/j.1365-2133.2007.08206.x

[11] Berthelot C, Dickerson MC, Rady P, He Q, Niroomand F, et al (2007) Treatment of a patient with epidermodysplasia verruciformis carrying a novel *EVER2* mutation with imiquimod. J Am Acad Dermatol 56: 882–886. 17368633 10.1016/j.jaad.2007.01.036

[12] Gober MD, Rady PL, He Q, Tucker SB, Tyring SK, et al (2007) Novel homozygous frameshift mutation of *EVER1* gene in an epidermodysplasia verruciformis patient. J Invest Dermatol 127: 817–820. 17139267 10.1038/sj.jid.5700641

[13] Zuo YG, Ma D, Zhang Y, Qiao J, Wang B (2006) Identification of a novel mutation and a genetic polymorphism of *EVER1* gene in two families with epidermodysplasia verruciformis. J Dermatol Sci 44: 153–159. 17008061 10.1016/j.jdermsci.2006.08.013

[14] Sun XK, Chen JF, Xu AE (2005) A homozygous nonsense mutation in the EVER2 gene leads to epidermodysplasia verruciformis. Clin Exp Dermatol 30: 573–574. 16045695 10.1111/j.1365-2230.2005.01858.x

[15] Tate G, Suzuki T, Kishimoto K, Mitsuya T (2004) Novel mutations of *EVER1/TMC6* gene in a Japanese patient with epidermodysplasia verruciformis. J Hum Genet 49: 223–225. 15042430 10.1007/s10038-004-0135-6

[16] Rady PL, De Oliveira WR, He Q, Festa C, Rivitti EA, et al (2007) Novel homozygous nonsense TMC8 mutation detected in patients with epidermodysplasia verruciformis from a Brazilian family. Br J Dermatol 157: 831–833. 17711520 10.1111/j.1365-2133.2007.08123.x

[17] Crequer A, Troeger A, Patin E, Ma CS, Picard C, et al. (2012) Human RHOH deficiency causes T cell defects and susceptibility to EV-HPV infections. Journal of Clinical Investigation in Press. 10.1172/JCI62949 PMC3428089 22850876

[18] Rogers HD, Macgregor JL, Nord KM, Tyring S, Rady P, et al (2009) Acquired epidermodysplasia verruciformis. J Am Acad Dermatol 60: 315–320. 19150275 10.1016/j.jaad.2008.08.035

[19] Daly ML, Hay RJ (2012) Epidermodysplasia verruciformis and human immunodeficiency virus infection: a distinct entity? Curr Opin Infect Dis. 10.1097/QCO.0b013e3283507fe7 22274729

[20] Lutzner MA (1985) Papillomavirus lesions in immunodepression and immunosuppression. Clin Dermatol 3: 165–169. 2850855 10.1016/0738-081x(85)90061-6

[21] Gross G, Ellinger K, Roussaki A, Fuchs PG, Peter HH, et al (1988) Epidermodysplasia verruciformis in a patient with Hodgkin’s disease: characterization of a new papillomavirus type and interferon treatment. J Invest Dermatol 91: 43–48. 2838553 10.1111/1523-1747.ep12463287

[22] Laffort C, Le Deist F, Favre M, Caillat-Zucman S, Radford-Weiss I, et al (2004) Severe cutaneous papillomavirus disease after haemopoietic stem-cell transplantation in patients with severe combined immune deficiency caused by common gammac cytokine receptor subunit or JAK-3 deficiency. Lancet 363: 2051–2054. 15207958 10.1016/S0140-6736(04)16457-X

[23] Byun M, Abhyankar A, Lelarge V, Plancoulaine S, Palanduz A, et al (2010) Whole-exome sequencing-based discovery of STIM1 deficiency in a child with fatal classic Kaposi sarcoma. J Exp Med 207: 2307–2312. 20876309 10.1084/jem.20101597 PMC2964585

[24] Bolze A, Byun M, McDonald D, Morgan NV, Abhyankar A, et al (2010) Whole-exome-sequencing-based discovery of human FADD deficiency. Am J Hum Genet 87: 873–881. 21109225 10.1016/j.ajhg.2010.10.028 PMC2997374

[25] Liu L, Okada S, Kong XF, Kreins AY, Cypowyj S, et al. Gain-of-function human STAT1 mutations impair IL-17 immunity and underlie chronic mucocutaneous candidiasis. J Exp Med 208: 1635–1648. 10.1084/jem.20110958 PMC3149226 21727188

[26] Romero P, Zippelius A, Kurth I, Pittet MJ, Touvrey C, et al (2007) Four functionally distinct populations of human effector-memory CD8+ T lymphocytes. J Immunol 178: 4112–4119. 17371966 10.4049/jimmunol.178.7.4112

[27] Eller MS, Maeda T, Magnoni C, Atwal D, Gilchrest BA (1997) Enhancement of DNA repair in human skin cells by thymidine dinucleotides: evidence for a p53-mediated mammalian SOS response. Proc Natl Acad Sci U S A 94: 12627–12632. 9356500 10.1073/pnas.94.23.12627 PMC25061

[28] Weeda G, Ma LB, van Ham RC, van der Eb AJ, Hoeijmakers JH (1991) Structure and expression of the human XPBC/ERCC-3 gene involved in DNA repair disorders xeroderma pigmentosum and Cockayne’s syndrome. Nucleic Acids Res 19: 6301–6308. 1956789 10.1093/nar/19.22.6301 PMC329143

[29] Oh KS, Khan SG, Jaspers NG, Raams A, Ueda T, et al (2006) Phenotypic heterogeneity in the XPB DNA helicase gene (ERCC3): xeroderma pigmentosum without and with Cockayne syndrome. Hum Mutat 27: 1092–1103. 16947863 10.1002/humu.20392

[30] Nehme NT, Pachlopnik Schmid J, Debeurme F, Andre-Schmutz I, Lim A, et al. (2011) MST1 mutations in autosomal recessive primary immunodeficiency characterized by defective naive T cells survival. Blood 10.1182/blood-2011-09-378364. 10.1182/blood-2011-09-378364 PMC3824282 22174160

[31] Abdollahpour H, Appaswamy G, Kotlarz D, Diestelhorst J, Beier R, et al. (2012) The phenotype of human STK4 deficiency. Blood 10.1182/blood-2011-09-378158. 10.1182/blood-2011-09-378158 PMC3325036 22294732

[32] Zhou D, Medoff BD, Chen L, Li L, Zhang XF, et al (2008) The Nore1B/Mst1 complex restrains antigen receptor-induced proliferation of naive T cells. Proc Natl Acad Sci U S A 105: 20321–20326. 19073936 10.1073/pnas.0810773105 PMC2600581

[33] Dong Y, Du X, Ye J, Han M, Xu T, et al (2009) A cell-intrinsic role for Mst1 in regulating thymocyte egress. J Immunol 183: 3865–3872. 19692642 10.4049/jimmunol.0900678

[34] Avruch J, Zhou D, Fitamant J, Bardeesy N (2011) Mst1/2 signalling to Yap: gatekeeper for liver size and tumour development. Br J Cancer 104: 24–32. 21102585 10.1038/sj.bjc.6606011 PMC3039822

[35] Lee JH, Kim TS, Yang TH, Koo BK, Oh SP, et al (2008) A crucial role of WW45 in developing epithelial tissues in the mouse. EMBO J 27: 1231–1242. 18369314 10.1038/emboj.2008.63 PMC2367404

[36] Schlegelmilch K, Mohseni M, Kirak O, Pruszak J, Rodriguez JR, et al (2011) Yap1 acts downstream of alpha-catenin to control epidermal proliferation. Cell 144: 782–795. 21376238 10.1016/j.cell.2011.02.031 PMC3237196

[37] Tan MJ, White EA, Sowa ME, Harper JW, Aster JC, et al (2012) Cutaneous beta-human papillomavirus E6 proteins bind Mastermind-like coactivators and repress Notch signaling. Proc Natl Acad Sci U S A 109: E1473–1480. 22547818 10.1073/pnas.1205991109 PMC3384212

[38] Abecasis GR, Cherny SS, Cookson WO, Cardon LR (2002) Merlin–rapid analysis of dense genetic maps using sparse gene flow trees. Nat Genet 30: 97–101. 11731797 10.1038/ng786

[39] Wang K, Chen Z, Tadesse MG, Glessner J, Grant SF, et al (2008) Modeling genetic inheritance of copy number variations. Nucleic Acids Res 36: e138. 18832372 10.1093/nar/gkn641 PMC2588508

[40] Li H, Durbin R (2009) Fast and accurate short read alignment with Burrows-Wheeler transform. Bioinformatics 25: 1754–1760. 19451168 10.1093/bioinformatics/btp324 PMC2705234

[41] McKenna A, Hanna M, Banks E, Sivachenko A, Cibulskis K, et al (2010) The Genome Analysis Toolkit: a MapReduce framework for analyzing next-generation DNA sequencing data. Genome Res 20: 1297–1303. 20644199 10.1101/gr.107524.110 PMC2928508

[42] Li H, Handsaker B, Wysoker A, Fennell T, Ruan J, et al (2009) The Sequence Alignment/Map format and SAMtools. Bioinformatics 25: 2078–2079. 19505943 10.1093/bioinformatics/btp352 PMC2723002

[43] Tunnacliffe A, Olsson C, de la Hera A (1989) The majority of human CD3 epitopes are conferred by the epsilon chain. Int Immunol 1: 546–550. 2484963 10.1093/intimm/1.5.546

[44] Andre-Schmutz I, Le Deist F, Hacein-Bey-Abina S, Vitetta E, Schindler J, et al (2002) Immune reconstitution without graft-versus-host disease after haemopoietic stem-cell transplantation: a phase 1/2 study. Lancet 360: 130–137. 12126823 10.1016/S0140-6736(02)09413-8

[45] de Saint Basile G, Geissmann F, Flori E, Uring-Lambert B, Soudais C, et al (2004) Severe combined immunodeficiency caused by deficiency in either the delta or the epsilon subunit of CD3. J Clin Invest 114: 1512–1517. 15546002 10.1172/JCI22588 PMC525745

[46] de Villartay JP, Lim A, Al-Mousa H, Dupont S, Dechanet-Merville J, et al (2005) A novel immunodeficiency associated with hypomorphic RAG1 mutations and CMV infection. J Clin Invest 115: 3291–3299. 16276422 10.1172/JCI25178 PMC1265866

[47] Lugada ES, Mermin J, Kaharuza F, Ulvestad E, Were W, et al (2004) Population-based hematologic and immunologic reference values for a healthy Ugandan population. Clin Diagn Lab Immunol 11: 29–34. 14715541 10.1128/CDLI.11.1.29-34.2004 PMC321349

[48] Shearer WT, Rosenblatt HM, Gelman RS, Oyomopito R, Plaeger S, et al (2003) Lymphocyte subsets in healthy children from birth through 18 years of age: the Pediatric AIDS Clinical Trials Group P1009 study. J Allergy Clin Immunol 112: 973–980. 14610491 10.1016/j.jaci.2003.07.003

[49] Bisset LR, Lung TL, Kaelin M, Ludwig E, Dubs RW (2004) Reference values for peripheral blood lymphocyte phenotypes applicable to the healthy adult population in Switzerland. Eur J Haematol 72: 203–212. 14962239 10.1046/j.0902-4441.2003.00199.x

[50] Eidenschenk C, Jouanguy E, Alcais A, Mention JJ, Pasquier B, et al (2006) Familial NK cell deficiency associated with impaired IL-2- and IL-15-dependent survival of lymphocytes. J Immunol 177: 8835–8843. 17142786 10.4049/jimmunol.177.12.8835

