# Supplementary figures and images for: Inherited MST1 Deficiency Underlies Susceptibility to EV-HPV Infections
Source: PLoS One. 2012 Aug 27;7(8):e44010. doi: 10.1371/journal.pone.0044010 (PMC3428299; doi:10.1371/journal.pone.0044010)

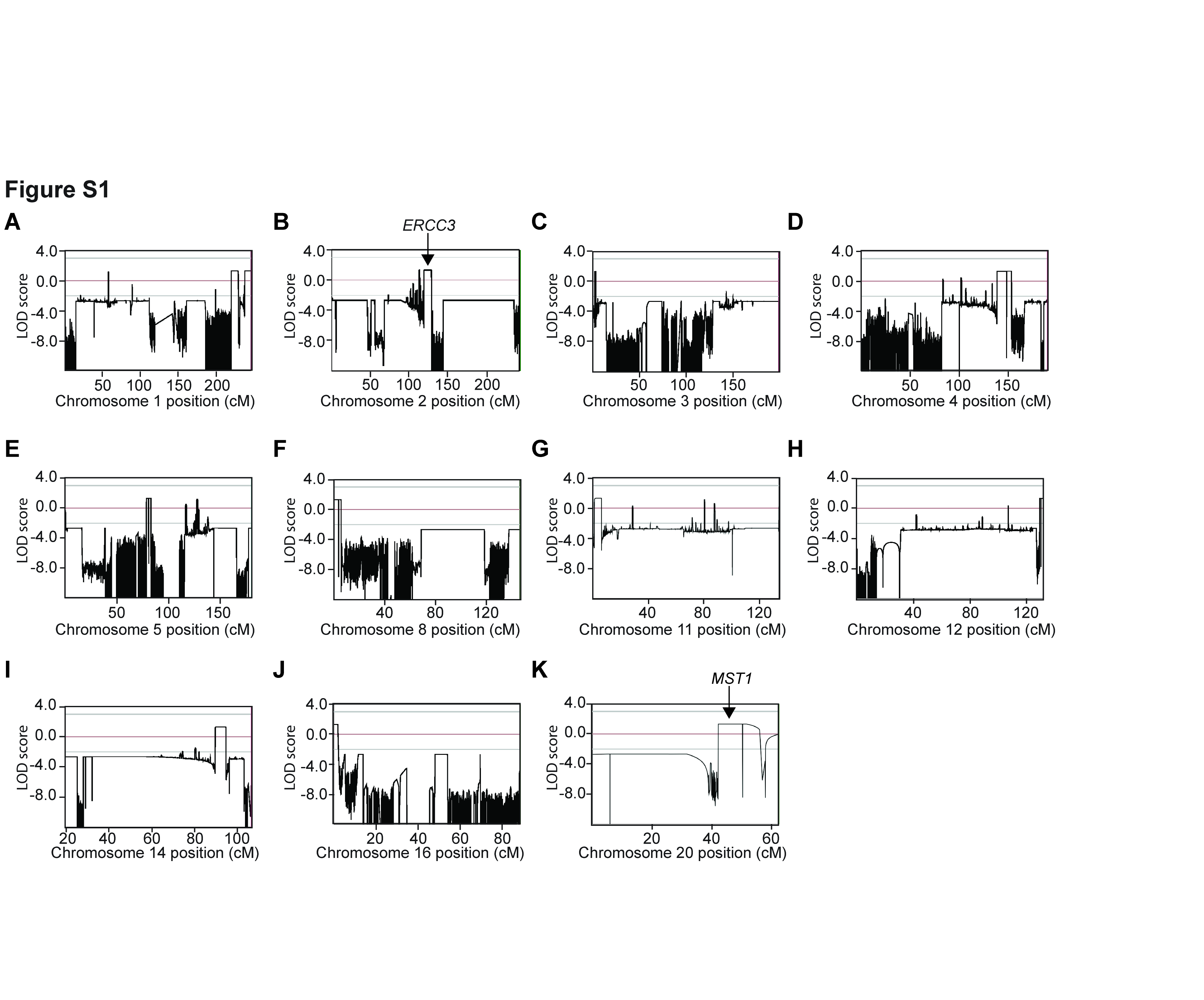

Supplement: Figure S1 — Analysis of multipoint linkage between the patient’s disease and chromosomes 1, 2, 3, 4, 5, 8, 11, 12, 14, 16, 20, with a full penetrance model. Only chromosomes including regions with a maximal LOD score are shown. LOD scores (Y axis) are plotted against chromosomal position (in cM). The locations of ERCC3 and MST1 are indicated by black arrows. (TIF) [file pone.0044010.s001.tif]
