# Supplementary material for: Inherited MST1 Deficiency Underlies Susceptibility to EV-HPV Infections
Source: PLoS One. 2012 Aug 27;7(8):e44010. doi: 10.1371/journal.pone.0044010 (PMC3428299; doi:10.1371/journal.pone.0044010)
Supplement: Table S1 — Humoral immunity of the patient’s peripheral blood before Ig substitution. (DOCX) [file pone.0044010.s002.docx]

**Table S1.** Humoral immunity of the patient’s peripheral blood before Ig substitution

| Patient’s age | 8 years | 13 years | 14 years | 17 years |
| --- | --- | --- | --- | --- |
| *Serum Ig (mg/ml) (normal range)* |  |  |  |  |
| IgG | 19.6 (8.3-14.3^A^) | 16.4 (5.5-10.2^A^) | 19.0 (4.8-14^A^) | 15.70 (4.8-14^A^) |
| IgA | 6.20 (1.02-1.94^A^) | 5.37 (0.41-1.41^A^) | 8.37 (0.49-1.90^A^) | 6.42 (0.49-1.90^A^) |
| IgM | 0.44 (0.68-1.28^A^) | 0.44 (0.54-1.53^A^) | 0.47 (0.55-1.77^A^) | 0.34 (0.55-1.77^A^) |
| IgE (kIU/l) | 3,609 (10-100^A^) | 5,598 (10-100 ^A^) | ND | ND |
| *Specific antibodies* |  |  |  |  |
| Tetanus, Poliovirus | ND | + | ND | ND |
| VZV | + (4500 mIU/ml) | + | ND | ND |
| MMR | ND | + | ND | ND |
| Rubella alone | + (>270 IU/ml) | ND | ND | ND |
| Allohemagglutinin titer IgG | ND | 1:1 | ND | ND |
| Diphtheria | ND | - | ND | ND |
| Pneumococcus | ND | ND | - | ND |
| Haemophilus | ND | ND | - | ND |
| *Autoantibodies (antinuclear antibodies) (cpm)* |  |  |  |  |
| U1ARNP (positive: >230) | ND | ND | ND | 144 |
| Ro60 (SSA) (positive: >225) | ND | ND | ND | 53 |
| SSB (positive: >180) | ND | ND | ND | 19 |

^A^ normal ranges from the work of Nehme *et al.* (1)

1. Nehme, N.T., Pachlopnik Schmid, J., Debeurme, F., Andre-Schmutz, I., Lim, A., Nitschke, P., Rieux-Laucat, F., Lutz, P., Picard, C., Mahlaoui, N., et al. 2011. MST1 mutations in autosomal recessive primary immunodeficiency characterized by defective naive T cells survival. *Blood* 10.1182/blood-2011-09-378364.
